# Modeling and multi-objective optimization of student engagement in virtual learning environments: a hybrid PDE–NSGA-II framework for digital music education

**DOI:** 10.3389/fpsyg.2026.1861905

**Published:** 2026-07-20

**Authors:** Xiaolei Qiao

**Affiliations:** College of Music and Education, Zhengzhou University of Industrial Technology, Xinzheng, China

**Keywords:** adaptive learning systems, digital music education, NSGA-II optimization, PDE modeling, student engagement

## Abstract

**Introduction:**

Student engagement is a key determinant of learning success in virtual learning environments, particularly in digital music education, where sustained practice and interaction are essential. This study proposes a hybrid framework that integrates partial differential equation (PDE) modeling and multi-objective optimization to analyze and improve learner engagement.

**Methods:**

Using the Predict Online Course Engagement Dataset, variables related to engagement, performance, and dropout were extracted and transformed into continuous temporal representations. A PDE-based model was developed to represent how student engagement changes over time, spreads across learning activities, and declines when participation decreases.

**Results:**

The PDE model showed stable convergence and effectively captured temporal engagement dynamics. Optimization revealed trade-offs among objectives, increasing engagement from 0.50 to 0.57 and 0.65, improving performance from 0.64 to 0.69 and 0.73, and reducing dropout risk from 0.36 to 0.30 and 0.27 under balanced and high-engagement strategies, respectively. Sensitivity analysis indicated that higher diffusion and lower decay rates improved outcomes.

**Discussion:**

The proposed PDE–NSGA-II framework provides a unified approach to adaptive learning support, offering practical guidance to enhance engagement, performance, and retention in digital music education.

## 1 Introduction

The rapid expansion of virtual learning environments has transformed educational delivery by enabling flexible, scalable, and technology-driven learning (Dritsas and Trigka, 2025; Makda, 2025; Cao et al., 2025). Despite these advancements, sustaining student engagement remains a fundamental challenge, particularly in domains such as digital music education, where learning depends on continuous practice, repetition, and active interaction (Shen, 2025; Huang C. et al., 2025; Shao et al., 2025). Engagement is strongly associated with performance and retention, and its inherently dynamic nature requires modeling approaches that capture temporal evolution rather than static observations (Alzahrani et al., 2025; Beyari, 2025; Chu et al., 2025). Recent research in learning analytics has increasingly focused on modeling and predicting student engagement using advanced data-driven techniques. Abedi and Khan (2021) employed deep learning methods to detect engagement from behavioral and visual data. Similarly, Salem et al. (2025) emphasized the importance of real-time disengagement detection in online learning systems. While these approaches successfully capture temporal patterns, they primarily focus on detection and prediction rather than on decision-making or optimization (Song et al., 2025; Xu et al., 2026).

Recent developments in Edu X.0 and Internet of Education (IoEd) research emphasize the transition from technology-enhanced learning toward intelligent, interconnected educational ecosystems that integrate data analytics, artificial intelligence, adaptive learning, and human-centered educational governance (Holmes and Miao, 2023; Bo, 2025; Mattiello et al., 2025a; Mattiello, 2024). Within this paradigm, educational systems are increasingly expected to support continuous learner monitoring, personalized interventions, competency development, and evidence-based decision-making. Learning analytics has emerged as a foundational component of this transformation by enabling the collection and interpretation of learner data to improve educational outcomes (Long and Siemens, 2014). The present study is situated within this broader Edu X.0 perspective and investigates how dynamic modeling and optimization techniques can contribute to intelligent educational adaptation.

Parallel developments in learning analytics highlight the role of temporal engagement patterns and early intervention strategies (Zhang and Li, 2025; Alalawi et al., 2025). Johnston et al. (2025) and Tu et al. (2026) demonstrated that engagement evolves over time and that early identification of disengagement is critical for improving outcomes. However, these approaches rely on discrete or statistical representations and do not incorporate continuous dynamic modeling. To address the need for more realistic representations, continuous-time modeling approaches have been explored (Zhang et al., 2025; Huang Y. et al., 2025). Differential equation-based models describe learning as a dynamic process that evolves over time, enabling analysis of engagement progression and interaction effects (Zeng, 2026; Fabregas et al., 2026). Although these models capture temporal behavior more effectively, they remain largely descriptive and lack optimization mechanisms to support adaptive decision-making.

In parallel, optimization techniques have been widely applied to educational problems. The Non-dominated Sorting Genetic Algorithm II, developed by Mishra et al. (2025) and Wang et al. (2026), has been used in recent studies to optimize learning pathways and resource allocation. Huang et al. (2023) explored AI-driven personalized learning systems that adapt to student behavior. However, these approaches typically operate on static or discrete data and lack integration with continuous engagement dynamics. In the field of music education, digital learning environments have gained increasing attention for their ability to support interactive, flexible learning. Recent studies such as Al-Rousan et al. (2025) and Yang and Li (2025) demonstrated that video-based instruction and digital tools significantly enhance student engagement and creativity. Xie et al. (2025) highlighted the role of interactive platforms in improving participation and skill development. However, these studies are primarily empirical and lack formal mathematical frameworks for modeling and optimizing engagement. A structured comparison of recent studies is presented in Table 1.

**Table 1:** Comparative analysis of recent studies on student engagement modeling and optimization in virtual learning environments, highlighting methodological approaches, temporal capabilities, applicability to music education, and distinctions from the proposed PDE–NSGA-II framework.

Study/approach	References	Modeling type	Optimization	Temporal dynamics	Music context	Compared to proposed work
Deep learning engagement detection	Abedi and Khan, 2021	Deep learning	No	Yes	No	Detection only, no optimization
Disengagement detection review	Salem et al., 2025	AI/review	No	Yes	No	Monitoring without decision support
Temporal learning analytics	Johnston et al., 2025	ML/statistical	No	Yes	No	No continuous modeling
AI-based personalized learning	Al-Rousan et al., 2025	AI-based	Partial	Yes	No	No mathematical dynamic model
Digital tools in music education	Song et al., 2025	Empirical	No	Partial	Yes	No optimization framework
Online music learning outcomes	Yang and Li, 2025	Experimental	No	Partial	Yes	Limited to evaluation
Interactive music platforms	Xie et al., 2025	Qualitative	No	Partial	Yes	No quantitative modeling
Proposed framework	This work	PDE-based dynamic	Yes (NSGA-II)	Yes	Yes	Integrated modeling and optimization

Table 1 shows that recent studies address individual aspects of the engagement problem but do not provide a unified framework. Deep learning and learning analytics approaches focus on detection and prediction, while optimization methods lack dynamic modeling. In music education, existing work emphasizes interaction and practice but remains largely empirical. The proposed framework addresses these gaps by integrating partial differential equation modeling with multi-objective optimization. This approach enables the representation of engagement as a continuous dynamic process while simultaneously identifying optimal learning strategies. The framework is further adapted to digital music education by linking engagement variables to practice behavior, video interaction, and skill development. From an Edu X.0 perspective, educational systems are increasingly characterized by intelligent connectivity, continuous data exchange, and AI-supported decision-making across learners, instructors, and institutional platforms. The Internet of Education (IoEd) extends traditional learning analytics by integrating sensing, modeling, and adaptive intervention mechanisms within interconnected educational ecosystems. In this context, the proposed PDE–NSGA-II framework is designed not only to model engagement dynamics but also to support intelligent adaptation and optimization processes that are central to next-generation higher education environments.

Although the adopted dataset contains standard indicators of learner behavior, the objective of this study extends beyond descriptive analysis and prediction. Student engagement is inherently dynamic and evolves continuously through interactions with learning activities. Conventional statistical and machine learning approaches generally treat observations as discrete instances, thereby providing limited insight into the temporal mechanisms governing the evolution of engagement. The PDE formulation is adopted to represent engagement as a continuous dynamic process, whereas NSGA-II is employed to identify optimal trade-offs among engagement, performance, and dropout risk. The combined framework, therefore, supports both dynamic analysis and optimization-driven instructional decision making.

While previous research has explored learning analytics, intelligent tutoring systems, educational AI, and digital transformation in higher education (Anderson et al., 1995; Koedinger et al., 2010; Zawacki-Richter et al., 2019; Castro Benavides et al., 2020), limited attention has been given to the integration of continuous engagement modeling and optimization within Edu X.0 and IoEd environments. The present study addresses this gap by combining dynamic engagement analysis with multi-objective decision support for adaptive educational systems. The main contributions are 3-fold. First, a continuous-time model is developed to capture the evolution of engagement. Second, a multi-objective optimization framework is introduced to balance engagement, performance, and dropout risk. Third, the approach is extended to music education, providing domain-specific insights into practice-driven learning. This integration advances the state of the art by combining dynamic modeling and optimization within a unified framework, while offering practical implications for adaptive and personalized digital music education systems.

## 2 Methodology and theoretical framework

### 2.1 Overall research workflow

The proposed methodology consists of four sequential stages. First, the Predict Online Course Engagement Dataset is collected and pre-processed through normalization and feature construction. Second, engagement-related variables are transformed into continuous temporal representations. Third, a PDE-based dynamic model is developed to simulate the temporal evolution of student engagement. Fourth, the outputs generated by the PDE model are incorporated into a multi-objective optimization framework based on NSGA-II to identify learning strategies that maximize engagement and performance while minimizing dropout risk.

**Figure 1:**
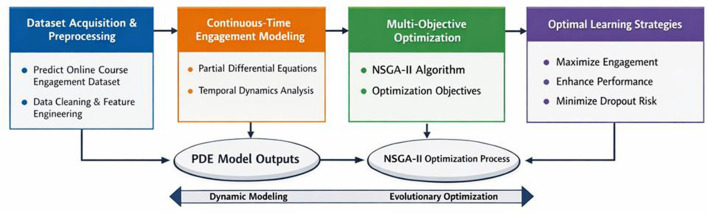
Schematic representation of the methodological framework, illustrating the sequential integration of dataset preprocessing, continuous-time engagement modeling based on partial differential equations, and multi-objective optimization using the NSGA-II algorithm to derive optimal learning strategies under competing performance criteria.

For reproducibility, the objective functions are evaluated directly from the PDE-generated engagement trajectories obtained for each candidate solution. The engagement objective is computed as the negative cumulative engagement over the simulation period, where the cumulative engagement is determined from the temporal and spatial integration of the engagement field E(t,s). The performance objective is computed as the negative performance score derived from the average engagement level predicted by the PDE model during the simulation horizon. The dropout objective is computed as the estimated dropout risk, which is determined from the proportion of low-engagement states observed in the simulated engagement trajectory. Consequently, for every candidate solution generated by NSGA-II, the PDE model is first solved numerically, after which the resulting engagement field is used to calculate engagement, performance, and dropout-risk values. These three quantities form the fitness vector employed during non-dominated sorting, crowding-distance calculation, and solution selection. Figure 1 summarizes the complete workflow and the information flow between each stage.

### 2.2 Dataset description and pre-processing

The Predict Online Course Engagement Dataset is adopted as the empirical foundation for the modeling framework because it provides structured observations of student interactions, learning activities, and performance outcomes in virtual learning environments. The dataset comprises quantitative variables reflecting behavioral engagement, including time investment, resource utilization, and assessment performance. These variables are transformed into a mathematically consistent form to support continuous-time modeling and subsequent optimization. Let *N* denote the total number of learners and *M*, the number of observed features. The dataset is represented as a matrix as Equation 1:


$$\begin{array}{l} \text{X}=\left(x_{i,j}\right)\in {\mathbb{R}}^{N\times M}, \end{array}$$ (1)

where *x*_*i,j*_ denotes the value of the *j*-th feature for the *i*-th learner. To ensure numerical stability and comparability across heterogeneous variables, min–max normalization is applied. Each feature is rescaled into the unit interval as Equation 2:


$$\begin{array}{l} {\tilde{x}}_{i,j}=\frac{x_{i,j}-\min\left(x_{j}\right)}{\max\left(x_{j}\right)-\min\left(x_{j}\right)}, \end{array}$$ (2)

where min(*x*_*j*_) and max(*x*_*j*_) denote the minimum and maximum values of the *j*-th feature, respectively. An aggregate engagement index is constructed to represent each learner' s overall behavioral state. This index is defined as a weighted combination of normalized features as Equation 3:


$$\begin{array}{l} E_{i}=\overset{M}{\underset{j=1}{\sum }}w_{j}{\tilde{x}}_{i,j}, \end{array}$$ (3)

where *w*_*j*_ represents the relative importance assigned to the feature *j*, subject to the constraint as Equation 4:


$$\begin{array}{l} \overset{M}{\underset{j=1}{\sum }}w_{j}=1. \end{array}$$ (4)

To enable continuous-time modeling, discrete observations are embedded into a temporal domain. Let *t* ∈ [0, *T*] denote the learning duration. The engagement trajectory of the learner *i* is approximated as a continuous function as Equation 5:


$$\begin{array}{l} E_{i}\left(t\right)=I\left(E_{i}^{\left(k\right)},t_{k}\right), \end{array}$$ (5)

where $I\left(E_{i}^{\left(k\right)},t_{k}\right)$ denotes an interpolation operator applied over discrete time points *t*_*k*_. The transformation from discrete observations to a continuous representation is motivated by the assumption that learner engagement evolves as a temporally continuous process rather than as isolated events. Individual interactions recorded at discrete time points are treated as samples from an underlying latent engagement trajectory. The interpolation operator, therefore, provides an approximation of this continuous state function, enabling the application of differential operators to characterize rates of change, diffusion effects, and long-term engagement dynamics. This continuous-state formulation is consistent with dynamical systems approaches that model behavioral evolution over time and provides the mathematical basis for subsequent PDE analysis.

Performance is represented through an outcome variable derived from assessment-related features. A normalized performance score is defined as Equation 6:


$$\begin{array}{l} P_{i}=\frac{1}{K}\overset{K}{\underset{k=1}{\sum }}s_{i,k}, \end{array}$$ (6)

where *s*_*i,k*_ denotes the score of the learner *i* in the *k*-th assessment and *K* is the total number of assessments. To capture dropout behavior, a binary variable is introduced as Equation 7:


$$\begin{array}{l} D_{i}=\{\begin{array}{cc} 1, & if\;learner\;i\;does\;not\;complete\;the\;course,\\ 0, & otherwise. \end{array} \end{array}$$ (7)

Finally, the preprocessed dataset is expressed as a structured state vector for each learner as Equation 8:


$$\begin{array}{l} {\text{z}}_{i}\left(t\right)=\left[E_{i}\left(t\right),P_{i},D_{i}\right], \end{array}$$ (8)

which serves as the foundational input for the subsequent dynamic modeling and optimization procedures.

### 2.3 Feature engineering and temporal representation

The preprocessed variables are further transformed to construct a set of informative features capturing the intensity, frequency, and progression of learner engagement over time. These transformations enable the representation of learning behavior as a continuous and evolving process, which is required for subsequent dynamic modeling. Let *E*_*i*_(*t*) denote the previously defined continuous engagement function. To capture the rate of change of engagement over time, the first-order temporal derivative is introduced as Equation 9:


$$\begin{array}{l} \frac{\partial E_{i}\left(t\right)}{\partial t}, \end{array}$$ (9)

which reflects the instantaneous variation in engagement and allows the identification of increasing or decreasing behavioral trends. To represent cumulative learning effort, a time-integrated engagement measure is defined as Equation 10:


$$\begin{array}{l} C_{i}\left(t\right)=\int_{0}^{t}E_{i}\left(\tau \right)d\tau , \end{array}$$ (10)

where *C*_*i*_(*t*) quantifies the total engagement accumulated up to time *t*. The variability of engagement is incorporated through a temporal variance measure, expressed as Equation 11:


$$\begin{array}{l} \sigma_{i}^{2}=\frac{1}{T}\int_{0}^{T}{\left(E_{i}\left(t\right)-{\overset{-}{E}}_{i}\right)}^{2}dt, \end{array}$$ (11)

where ${\overset{-}{E}}_{i}$ denotes the mean engagement level over the interval [0, *T*]. This measure captures fluctuations in learner activity and distinguishes stable from irregular engagement patterns. To account for the intensity of interactions across multiple learning activities, a composite activity function is defined. Let ${\tilde{x}}_{i,j}\left(t\right)$ denote the time-dependent representation of feature *j*. The aggregated activity level is expressed as Equation 12:


$$\begin{array}{l} A_{i}\left(t\right)=\overset{M}{\underset{j=1}{\sum }}\alpha_{j}{\tilde{x}}_{i,j}\left(t\right), \end{array}$$ (12)

where α_*j*_ represents feature-specific scaling coefficients that reflect each activity type' s contribution. A normalized temporal engagement profile is then constructed to ensure consistency across learners as Equation 13:


$$\begin{array}{l} {Ê}_{i}\left(t\right)=\frac{E_{i}\left(t\right)}{\underset{t\in \left[0,T\right]}{\max}E_{i}\left(t\right)}, \end{array}$$ (13)

which bounds engagement within a standardized range, facilitating comparative analysis. To characterize the persistence of engagement, a decay-adjusted formulation is introduced as Equation 14:


$$\begin{array}{l} E_{i}^{*}\left(t\right)=E_{i}\left(t\right)e^{-\lambda t}, \end{array}$$ (14)

where λ > 0 represents a temporal decay parameter that models diminishing attention or motivation over time. Finally, the temporal feature space is defined as a composite representation as Equation 15:


$$\begin{array}{l} {\text{f}}_{i}\left(t\right)=\left[{Ê}_{i}\left(t\right),\;\frac{\partial E_{i}\left(t\right)}{\partial t},\;C_{i}\left(t\right),\;\sigma_{i}^{2},\;A_{i}\left(t\right),\;E_{i}^{*}\left(t\right)\right], \end{array}$$ (15)

which encapsulates both instantaneous and cumulative aspects of learner behavior. This representation serves as the input for the subsequent formulation of the dynamic engagement model.

### 2.4 Mathematical modeling of student engagement using PDEs

The temporal feature representation is formulated as a continuous-time dynamical system to describe the evolution of student engagement over time and across learning activities. Engagement is treated as a distributed quantity that evolves under the combined effects of temporal progression and interaction diffusion.

Let *E*_*i*_(*t*) denote the engagement level of the learner *i* at time *t*. The general form of the governing equation is defined as Equation 16:


$$\begin{array}{l} \frac{\partial E_{i}\left(t\right)}{\partial t}=F\left(E_{i}\left(t\right),{\text{f}}_{i}\left(t\right)\right), \end{array}$$ (16)

where F represents a functional operator that captures the influence of behavioral features on engagement dynamics.

To incorporate diffusion effects across learning activities, a spatial dimension is introduced over the feature space. Let *x* ∈ Ω ⊂ ℝ denote an abstract activity domain. The engagement function is extended to *E*_*i*_(*x, t*), and the model is expressed as a diffusion-reaction equation as Equation 17:


$$\begin{array}{l} \frac{\partial E_{i}\left(x,t\right)}{\partial t}=D\frac{\partial^{2}E_{i}\left(x,t\right)}{\partial x^{2}}+R\left(E_{i}\left(x,t\right),{\text{f}}_{i}\left(t\right)\right), \end{array}$$ (17)

where *D* > 0 is the diffusion coefficient and *R* is a reaction term that represents changes in local engagement.

The reaction component is defined to account for growth and decay mechanisms as Equation 18:


$$\begin{array}{l} R\left(E_{i}\left(x,t\right),{\text{f}}_{i}\left(t\right)\right)=\beta_{1}A_{i}\left(t\right)+\beta_{2}P_{i}-\beta_{3}E_{i}\left(x,t\right), \end{array}$$ (18)

where β_1_, β_2_, β_3_ are positive parameters controlling the influence of activity intensity, performance, and natural decay, respectively.

Substituting the reaction term into the governing equation yields as Equation 19:


$$\begin{array}{l} \frac{\partial E_{i}\left(x,t\right)}{\partial t}=D\frac{\partial^{2}E_{i}\left(x,t\right)}{\partial x^{2}}+\beta_{1}A_{i}\left(t\right)+\beta_{2}P_{i}-\beta_{3}E_{i}\left(x,t\right). \end{array}$$ (19)

The initial condition is specified as Equation 20:


$$\begin{array}{l} E_{i}\left(x,0\right)=E_{i}^{0}\left(x\right), \end{array}$$ (20)

where $E_{i}^{0}\left(x\right)$ denotes the initial engagement distribution.

Boundary conditions are imposed to ensure that the problem is well-posed. A no-flux condition is adopted as Equation 21:


$$\begin{array}{l} \frac{\partial E_{i}\left(x,t\right)}{\partial x}{|}_{x\in \partial \Omega }=0, \end{array}$$ (21)

which implies that engagement remains within the defined activity domain.

To incorporate dropout influence, a damping term is introduced as Equation 22:


$$\begin{array}{l} -\gamma D_{i}E_{i}\left(x,t\right), \end{array}$$ (22)

where γ > 0 is a dropout sensitivity parameter. The complete model becomes as Equation 23:


$$\begin{array}{r} \frac{\partial E_{i}\left(x,t\right)}{\partial t}=D\frac{\partial^{2}E_{i}\left(x,t\right)}{\partial x^{2}}+\beta_{1}A_{i}\left(t\right)+\beta_{2}P_{i}-\beta_{3}E_{i}\left(x,t\right)\\ -\gamma D_{i}E_{i}\left(x,t\right). \end{array}$$ (23)

For analytical tractability, a steady-state solution is defined as Equation 24:


$$\begin{array}{l} \frac{\partial E_{i}\left(x,t\right)}{\partial t}=0, \end{array}$$ (24)

which leads as Equation 25:


$$\begin{array}{l} D\frac{\partial^{2}E_{i}\left(x\right)}{\partial x^{2}}+\beta_{1}A_{i}+\beta_{2}P_{i}-\left(\beta_{3}+\gamma D_{i}\right)E_{i}\left(x\right)=0. \end{array}$$ (25)

This formulation provides a continuous representation of engagement dynamics, where temporal evolution, activity diffusion, and performance interactions are jointly modeled within a unified partial differential equation framework.

In this study, engagement dynamics refer to temporal changes in learner participation and interaction behavior. Diffusion represents the propagation of engagement across related learning activities and resources, reflecting how activity in one learning component may influence engagement in others. Decay represents the gradual reduction of engagement over time due to diminishing attention, motivation, or participation. These definitions provide the operational interpretation of the PDE model components used throughout the analysis.

### 2.5 Multi-objective optimization using NSGA-II

In the present study, the decision vector is defined as **x** = [*x*_1_, *x*_2_, *x*_3_, *x*_4_], where *x*_1_ represents activity allocation intensity, *x*_2_ denotes interaction frequency within the virtual learning environment, *x*_3_ corresponds to the utilization weight assigned to learning resources, and *x*_4_ represents practice repetition intensity. All decision variables are normalized to the interval [0, 1] to ensure numerical consistency and comparability during the optimization process. These variables are selected because they directly influence the engagement dynamics described by the PDE model and can be adjusted by the NSGA-II algorithm to identify optimal learning strategies under competing objectives.

The dynamic engagement model is incorporated into a multi-objective optimization framework to identify optimal learning strategies under competing criteria. The optimization problem is formulated to simultaneously maximize engagement and academic performance while minimizing dropout risk, subject to the governing equations and their constraints. Let ${\text{u}}_{i}\in {\mathbb{R}}^{d}$ denote the vector of decision variables associated with the learner *i*, which may include controllable factors such as activity allocation, study intensity, or resource usage weights. The multi-objective problem is defined as Equation 26:


$$\begin{array}{l} \underset{{\text{u}}_{i}}{\min}\;\text{F}\left({\text{u}}_{i}\right)=\left[f_{1}\left({\text{u}}_{i}\right),\;f_{2}\left({\text{u}}_{i}\right),\;f_{3}\left({\text{u}}_{i}\right)\right], \end{array}$$ (26)

where each objective function corresponds to a specific learning criterion. The first objective is defined to maximize engagement. Since NSGA-II is formulated as a minimization procedure, the objective is expressed as Equation 27:


$$\begin{array}{l} f_{1}\left({\text{u}}_{i}\right)=-\int_{0}^{T}E_{i}\left(x,t;{\text{u}}_{i}\right)dt, \end{array}$$ (27)

which represents the negative cumulative engagement over the learning period. The second objective represents academic performance and is defined as Equation 28:


$$\begin{array}{l} f_{2}\left({\text{u}}_{i}\right)=-P_{i}\left({\text{u}}_{i}\right), \end{array}$$ (28)

where *P*_*i*_(**u**_*i*_) is the performance score influenced by the decision variables. The third objective minimizes dropout risk and is expressed as Equation 29:


$$\begin{array}{l} f_{3}\left({\text{u}}_{i}\right)=D_{i}\left({\text{u}}_{i}\right), \end{array}$$ (29)

where *D*_*i*_(**u**_*i*_) ∈ [0, 1] represents the probability of dropout. The optimization problem is subject to the constraint imposed by the PDE model as Equation 30:


$$\begin{array}{r} \frac{\partial E_{i}\left(x,t;{\text{u}}_{i}\right)}{\partial t}=D\frac{\partial^{2}E_{i}\left(x,t;{\text{u}}_{i}\right)}{\partial x^{2}}+\beta_{1}A_{i}\left(t;{\text{u}}_{i}\right)+\beta_{2}P_{i}\left({\text{u}}_{i}\right)\\ -\beta_{3}E_{i}\left(x,t;{\text{u}}_{i}\right)-\gamma D_{i}\left({\text{u}}_{i}\right)E_{i}\left(x,t;{\text{u}}_{i}\right). \end{array}$$ (30)

Additional feasibility constraints are imposed on the decision variables as Equation 31:


$$\begin{array}{l} {\text{u}}_{i}^{\min}\leq {\text{u}}_{i}\leq {\text{u}}_{i}^{\max}. \end{array}$$ (31)

The resulting optimization problem is therefore defined as a constrained multi-objective minimization task in which the objective vector *F*(*x*) = [*f*_1_(*x*), *f*_2_(*x*), *f*_3_(*x*)] is optimized subject to both the governing PDE dynamics and the admissible bounds of the decision variables. Specifically, the optimization seeks decision vectors that simultaneously maximize cumulative engagement and academic performance while minimizing dropout risk, under the constraint that the engagement state evolution satisfies the PDE model and all decision variables remain within the normalized interval [0, 1]. Consequently, the feasible search space is determined by the combined effects of dynamic system constraints and variable bounds, and only solutions that satisfy these conditions are considered during the NSGA-II evaluation and selection process.

The NSGA-II procedure operates on a population of candidate solutions. Let $P^{\left(g\right)}=\left({\text{u}}_{1},{\text{u}}_{2},\ldots ,{\text{u}}_{N}\right)$ denote the population at generation *g*. Individuals are evaluated using the objective vector **F**(**u**_*i*_), and non-dominated sorting is applied to partition the population into Pareto fronts as Equation 32:


$$\begin{array}{l} P^{\left(g\right)}=\overset{K}{\underset{k=1}{⋃}}F_{k}, \end{array}$$ (32)

where $F_{k}$ denotes the *k*-th non-dominated front. Crowding distance is computed to preserve diversity within each front. For objective *m*, the crowding distance of the solution *i* is given as Equation 33:


$$\begin{array}{l} d_{i}^{\left(m\right)}=\frac{f_{m}^{i+1}-f_{m}^{i-1}}{f_{m}^{\max}-f_{m}^{\min}}, \end{array}$$ (33)

and the total crowding distance is as Equation 34:


$$\begin{array}{l} d_{i}=\overset{3}{\underset{m=1}{\sum }}d_{i}^{\left(m\right)}. \end{array}$$ (34)

Selection is performed using a binary tournament based on rank and crowding distance. Offspring are generated through crossover and mutation operators as Equation 35, 36:


$$\begin{array}{l} u_{i}^{\;\prime }=C(u_{p},u_{q}), \end{array}$$ (35)

$$\begin{array}{l} u_{i}^{\;\prime \prime }=ℳ(u_{i}^{\;\prime \prime }), \end{array}$$ (36)

where C and M denote crossover and mutation, respectively. The population is iteratively updated until convergence, resulting in a Pareto-optimal set defined as Equation 37:


$$\begin{array}{l} P^{*}=\left({\text{u}}_{i}|∄\;{\text{u}}_{j}\;such\;that\;\text{F}\left({\text{u}}_{j}\right)≺\text{F}\left({\text{u}}_{i}\right)\right). \end{array}$$ (37)

This formulation enables the identification of optimal trade-offs between engagement, performance, and dropout risk within a unified multi-objective optimization framework.

### 2.6 Integration of PDE model with NSGA-II

The proposed framework establishes a direct connection between empirical data, dynamic engagement modeling, and optimization. The pre-processed dataset provides the initial engagement states and activity features used in the PDE formulation. The resulting engagement trajectories are subsequently employed to compute optimization objectives and evaluation metrics, thereby ensuring a unified analytical pipeline. The integration of the dynamic engagement model with the multi-objective optimization framework is achieved through a coupled evaluation mechanism, in which the solution of the partial differential equation governs the fitness assessment of candidate solutions. This coupling ensures that the temporal evolution of engagement directly influences the optimization process.

The integration procedure consists of five sequential stages. First, the engagement dataset is pre-processed and transformed into normalized temporal features. Second, these features are used to construct the initial engagement distribution and activity-related variables required by the PDE model. Third, each candidate solution generated by NSGA-II specifies a unique combination of decision variables controlling activity allocation, interaction frequency, resource utilization, and practice intensity. Fourth, the PDE model is solved numerically for each candidate solution to generate engagement trajectories over the learning period. Finally, the resulting engagement profiles are used to compute engagement, performance, and dropout-risk objective values, which are subsequently evaluated via Pareto-based optimization. This procedure establishes a direct connection among empirical observations, dynamic engagement modeling, and optimization-based decision-making. Within the Internet of Education (IoEd) paradigm, the proposed framework can be interpreted as a multi-layer educational intelligence architecture. The engagement dataset functions as the educational sensing layer, continuously capturing learner interaction data. The PDE component serves as the dynamic learner-state modeling layer, representing temporal changes in engagement. The NSGA-II module serves as the decision-optimization layer, identifying intervention strategies that balance engagement, performance, and retention objectives. This layered structure extends beyond engagement prediction by supporting intelligent educational decision-making within interconnected learning ecosystems.

Let **u**_*i*_ denote a candidate solution in the decision space. For each **u**_*i*_, the engagement dynamics are obtained by solving the governing equation subject to the corresponding parameter configuration. The resulting engagement field is expressed as Equation 38:


$$\begin{array}{l} E_{i}\left(x,t;{\text{u}}_{i}\right)=S\left({\text{u}}_{i}\right), \end{array}$$ (38)

where S denotes the solution operator associated with the PDE system. The fitness evaluation of everyone is therefore dependent on the computed engagement trajectory. The objective vector is reformulated as Equation 39:


$$\begin{array}{l} \text{F}\left({\text{u}}_{i}\right)=\text{G}\left(E_{i}\left(x,t;{\text{u}}_{i}\right),P_{i}\left({\text{u}}_{i}\right),D_{i}\left({\text{u}}_{i}\right)\right), \end{array}$$ (39)

where **G** maps the PDE-derived quantities to the optimization objectives. The objective functions are evaluated directly from the PDE-generated engagement trajectories. The cumulative engagement objective is computed from the integrated engagement field over the temporal and activity domains. The performance objective is estimated from the average engagement level obtained during the simulation period, reflecting the positive relationship between sustained engagement and academic achievement. The dropout objective is derived from the proportion of low-engagement states observed during the simulation and represents the likelihood of learner disengagement. Consequently, all optimization objectives are derived from the PDE solution, ensuring that candidate strategies are evaluated based on their dynamic effects on engagement behavior.

The PDE model and NSGA-II optimizer are coupled through a nested evaluation mechanism. For each candidate solution generated by NSGA-II, the PDE system is solved numerically to obtain engagement trajectories. These trajectories are then transformed into objective values that guide the optimization process. Consequently, optimization decisions are directly governed by the dynamic behavior predicted by the PDE model. Algorithm 1. PDE–NSGA-II Integration Procedure is as follows;

1. Initialize NSGA-II population.
2. Generate candidate decision vector x.
3. Assign x to PDE parameters and learning control variables.
4. Solve the PDE model numerically.
5. Compute engagement trajectory E(t,s).
6. Derive objective values:
  ° engagement score
  ° performance score
  ° dropout risk
7. Evaluate fitness vector F(x).
8. Perform non-dominated sorting and crowding-distance calculation.
9. Apply crossover and mutation operators.
10. Repeat until convergence.
11. Return Pareto-optimal solution set.

To ensure numerical implementation, the continuous domain is discretized. Let the temporal domain be partitioned into *N*_*t*_ steps and the spatial domain into *N*_*x*_ nodes. The discretized engagement field is defined as Equation 40:


$$\begin{array}{l} E_{i}^{k,n}\approx E_{i}\left(x_{n},t_{k};{\text{u}}_{i}\right), \end{array}$$ (40)

where *t*_*k*_ and *x*_*n*_ denote discrete-time and discrete-space points, respectively. A finite difference approximation is adopted for the temporal derivative as Equation 41:


$$\begin{array}{l} \frac{\partial E_{i}}{\partial t}\approx \frac{E_{i}^{k+1,n}-E_{i}^{k,n}}{\Delta t}, \end{array}$$ (41)

and for the spatial diffusion term as Equation 42:


$$\begin{array}{l} \frac{\partial^{2}E_{i}}{\partial x^{2}}\approx \frac{E_{i}^{k,n+1}-2E_{i}^{k,n}+E_{i}^{k,n-1}}{\Delta x^{2}} \end{array}$$ (42)

Substituting these approximations into the governing equation yields the discrete update scheme as Equation 43:


$$\begin{array}{r} E_{i}^{k+1,n}=E_{i}^{k,n}\\ +\Delta t[D\frac{E_{i}^{k,n+1}-2E_{i}^{k,n}+E_{i}^{k,n-1}}{\Delta x^{2}}+\beta_{1}A_{i}^{k}+\beta_{2}P_{i}\\ -\beta_{3}E_{i}^{k,n}-\gamma D_{i}E_{i}^{k,n}] \end{array}$$ (43)

This iterative scheme is applied for each candidate solution during the evaluation phase of the optimization algorithm. The resulting engagement trajectories are then used to compute the previously defined objective values. To ensure stability of the numerical solution, the discretization parameters satisfy the condition as Equation 44:


$$\begin{array}{l} \frac{D\Delta t}{\Delta x^{2}}\leq \frac{1}{2} \end{array}$$ (44)

which guarantees the convergence of the finite-difference scheme. The integration procedure is thus defined as a nested process in which each generation of the NSGA-II algorithm requires the numerical solution of the PDE system for all candidate solutions. Algorithmically, the framework operates as a nested optimization process. For each generation of the NSGA-II algorithm, a population of candidate decision vectors is generated and evaluated. The PDE model is solved for every candidate solution, producing a corresponding engagement trajectory. The resulting engagement dynamics are transformed into objective values and used for non-dominated sorting and crowding-distance calculations. Candidate solutions are then updated through crossover and mutation operators until convergence is achieved. The final Pareto-optimal set, therefore, represents learning strategies that satisfy the dynamic constraints imposed by the engagement model while balancing multiple educational objectives.

The optimized solution set is obtained as Equation 45:


$$\begin{array}{l} P^{*}=\arg\underset{{\text{u}}_{i}}{\min}\text{F}\left({\text{u}}_{i}\right), \end{array}$$ (45)

subject to the dynamic constraints imposed by the discretized engagement model.

### 2.7 Model implementation and computational setup

The proposed framework is implemented within a numerical computing environment that supports matrix operations, evolutionary algorithms, and the numerical solution of partial differential equations. The implementation is structured as a sequential pipeline in which data preprocessing, dynamic modeling, and optimization are executed in an integrated manner. Let the computational domain be discretized into *N*_*x*_ spatial nodes and *N*_*t*_ temporal steps. The total computational complexity associated with the PDE solver for a single candidate solution is expressed as Equation 46:


$$\begin{array}{l} C_{PDE}=O\left(N_{x}\cdot N_{t}\right), \end{array}$$ (46)

which reflects the iterative update of the engagement field across space and time. For the optimization component, let *N*_*p*_ denote the population size and *G* the number of generations. The overall computational cost of the NSGA-II procedure is given as Equation 47:


$$\begin{array}{l} C_{NSGA-II}=O\left(G\cdot N_{p}\cdot C_{PDE}\right), \end{array}$$ (47)

since everyone requires a full PDE evaluation during fitness computation. The initialization of the population is performed by sampling decision variables within predefined bound as Equation 48:


$$\begin{array}{l} {\text{u}}_{i}^{\left(0\right)}\sim U\left({\text{u}}^{\min},{\text{u}}^{\max}\right) \end{array}$$ (48)

where U denotes a uniform distribution over the feasible space. Algorithmic parameters are defined to ensure convergence and solution diversity. The crossover and mutation probabilities are denoted as *p*_*c*_ and *p*_*m*_, respectively.

To facilitate reproducibility, all computational experiments were conducted using a fixed set of initialization procedures and optimization parameters. Decision variables were normalized to the interval [0, 1], and candidate solutions were generated using uniform random sampling within the feasible search space. The same population initialization strategy, objective evaluation procedure, and convergence criteria were applied across all experimental runs. The PDE solver employed a consistent discretization scheme with fixed temporal and spatial resolutions satisfying the previously defined stability condition. Furthermore, identical parameter settings were maintained throughout all simulations to ensure comparability of results and repeatability of the optimization process.

The computational evaluation procedure follows a fixed workflow. For each candidate solution generated by NSGA-II, the corresponding decision variables are assigned to the PDE model parameters and control variables. The PDE system is then solved over the complete simulation horizon using the explicit finite-difference scheme described in Section 2.6. The resulting engagement trajectory is subsequently used to calculate the engagement, performance, and dropout objectives. After objective evaluation, non-dominated sorting and crowding-distance calculations are performed, followed by crossover and mutation operations to generate the next population. This evaluation process is repeated until the convergence criterion is satisfied. The optimization experiments employ a population size of 100 individuals, 200 generations, a crossover probability of 0.90, and a mutation probability of 0.10. All experiments use identical parameter settings and random initialization procedures to ensure comparability and repeatability of results.

The expected number of offspring generated per generation is expressed as Equations 49, 50:


$$\begin{array}{l} N_{offspring}=N_{p}\cdot p_{c} \end{array}$$ (49)

while mutation introduces stochastic perturbations to maintain exploration:


$$\begin{array}{l} {\text{u}}_{i}^{mut}={\text{u}}_{i}+\epsilon ,\epsilon \sim N\left(0,\sigma^{2}\right) \end{array}$$ (50)

The numerical solution of the PDE system is implemented using an explicit finite difference scheme, with a time step Δ*t* and spatial step Δ*x* selected to satisfy stability constraints defined previously. The total number of update operations required for one generation is therefore as Equation 51:


$$\begin{array}{l} N_{updates}=N_{p}\cdot N_{x}\cdot N_{t} \end{array}$$ (51)

To monitor convergence, a generational improvement metric is defined based on the change in objective values as Equation 52:


$$\begin{array}{l} \Delta {\text{F}}^{\left(g\right)}=\frac{1}{N_{p}}\overset{N_{p}}{\underset{i=1}{\sum }}∥{\text{F}}_{i}^{\left(g\right)}-{\text{F}}_{i}^{\left(g-1\right)}∥ \end{array}$$ (52)

where ∥ · ∥ denotes the Euclidean norm. Convergence is assumed when


$$\begin{array}{l} \Delta {\text{F}}^{\left(g\right)}<\varepsilon \end{array}$$ (53)

for a predefined tolerance ε > 0 as Equation 53. The final output of the computational procedure is the Pareto-optimal solution set obtained after *G*generations, together with the corresponding engagement trajectories and performance indicators. This implementation ensures a consistent integration of numerical simulation and evolutionary optimization within a scalable computational framework.

### 2.8 Evaluation metrics and validation strategy

The performance of the proposed framework is evaluated through a set of quantitative metrics that assess engagement dynamics, predictive consistency, and optimization effectiveness. The evaluation is conducted based on the objective functions and the resulting Pareto-optimal solutions, ensuring that both modeling accuracy and optimization quality are assessed. The evaluation metrics are mathematically defined to assess both model behavior and optimization performance. Engagement quality is measured through average engagement levels, prediction accuracy is evaluated using mean squared error, classification performance is assessed through dropout prediction accuracy, and optimization quality is examined using hypervolume and spacing indicators.

Let *E*_*i*_(*x, t*) denote the computed engagement field and *P*_*i*_, *D*_*i*_ the associated performance and dropout indicators. An average engagement measure over the temporal domain is defined as Equation 54:


$$\begin{array}{l} {\overset{-}{E}}_{i}=\frac{1}{T}\int_{0}^{T}E_{i}\left(t\right)dt \end{array}$$ (54)

which provides a global indicator of sustained learner activity. To evaluate prediction accuracy for outcome variables, a mean squared error metric is introduced as Equation 55:


$$\begin{array}{l} MSE=\frac{1}{N}\overset{N}{\underset{i=1}{\sum }}{\left(y_{i}-{ŷ}_{i}\right)}^{2} \end{array}$$ (55)

where *y*_*i*_ and ŷ_*i*_ denote observed and estimated values, respectively. For classification-related evaluation of dropout behavior, accuracy is defined as Equation 56:


$$\begin{array}{l} Accuracy=\frac{1}{N}\overset{N}{\underset{i=1}{\sum }}𝕀\left(D_{i}={\hat{D}}_{i}\right) \end{array}$$ (56)

where 𝕀 is the indicator function. The quality of the Pareto front obtained from the optimization process is assessed using the hypervolume indicator as Equation 57:


$$\begin{array}{l} HV=\lambda \left(\overset{N_{p}}{\underset{i=1}{⋃}}v_{i}\right) \end{array}$$ (57)

where *v*_*i*_ represents the dominated region corresponding to the solution *i*, and λ denotes the Lebesgue measure. To evaluate the spread and diversity of solutions, the spacing metric is defined as Equation 58:


$$\begin{array}{l} S=\sqrt{\frac{1}{N_{p}-1}\overset{N_{p}}{\underset{i=1}{\sum }}{\left(d_{i}-\overset{-}{d}\right)}^{2}} \end{array}$$ (58)

where *d*_*i*_ is the distance between neighboring solutions in objective space and $\overset{-}{d}$ is the mean distance. A cross-validation procedure is adopted to ensure robustness. Let the dataset be partitioned into *K* folds. The validation score is computed as Equation 59:


$$\begin{array}{l} CV=\frac{1}{K}\overset{K}{\underset{k=1}{\sum }}L^{\left(k\right)} \end{array}$$ (59)

where $L^{\left(k\right)}$ denotes the loss evaluated on the *k*-th fold. Sensitivity analysis is conducted to assess the influence of model parameters. For a parameter θ, sensitivity is defined as Equation 60:


$$\begin{array}{l} S_{\theta }=\frac{\partial \text{F}}{\partial \theta } \end{array}$$ (60)

which quantifies the variation in objective values with respect to changes in parameters. These evaluation measures provide a comprehensive validation framework, ensuring that the proposed model is assessed for accuracy, stability, and optimization performance across varying conditions. To facilitate replication, the complete evaluation process consists of data preprocessing, PDE simulation, objective computation, Pareto-based optimization, and metric calculation using the same parameter configuration reported throughout the study.

### 2.9 Adaptation to music education context

The proposed framework is extended to digital music education environments through a structured mapping between general engagement variables and domain-specific learning behaviors. Online music education is characterized by repeated interaction with video-based instructional content, practice-oriented learning, and progressive skill acquisition, which aligns with the behavioral patterns captured in the dataset. Let *E*_*i*_(*t*) denote the engagement level previously defined. In the context of music education, this quantity is interpreted as a composite measure of practice intensity, video interaction, and task repetition. A domain-specific engagement function is therefore defined as Equation 61:


$$\begin{array}{l} E_{i}^{\left(m\right)}\left(t\right)=\eta_{1}V_{i}\left(t\right)+\eta_{2}R_{i}\left(t\right)+\eta_{3}Q_{i}\left(t\right) \end{array}$$ (61)

where *V*_*i*_(*t*) represents video interaction, *R*_*i*_(*t*) denotes practice repetition, and *Q*_*i*_(*t*) corresponds to an assessment or quiz activity. The coefficients η_1_, η_2_, η_3_ satisfy as Equation 62:


$$\begin{array}{l} \eta_{1}+\eta_{2}+\eta_{3}=1 \end{array}$$ (62)

Skill development in music learning is inherently cumulative and is modeled as a function of engagement over time. A skill acquisition function is defined as Equation 63:


$$\begin{array}{l} S_{i}\left(t\right)=\int_{0}^{t}E_{i}^{\left(m\right)}\left(\tau \right)d\tau \end{array}$$ (63)

which reflects the accumulation of practice and learning effort. To account for diminishing returns in repetitive practice, a saturation effect is introduced as Equation 64:


$$\begin{array}{l} S_{i}^{*}\left(t\right)=\frac{S_{i}\left(t\right)}{1+\kappa S_{i}\left(t\right)} \end{array}$$ (64)

where κ > 0 is a saturation parameter that limits unbounded growth. Performance in music education is associated with skill proficiency and is expressed as Equation 65:


$$\begin{array}{l} P_{i}^{\left(m\right)}=\phi S_{i}^{*}\left(T\right) \end{array}$$ (65)

where ϕ is a scaling constant and *T* denotes the learning duration. Dropout behavior in music learning environments is often associated with reduced consistency in practice. A domain-specific dropout indicator is defined as Equation 66:


$$\begin{array}{l} D_{i}^{\left(m\right)}=\{\begin{array}{cc} 1, & if\;\frac{1}{T}\int_{0}^{T}E_{i}^{\left(m\right)}\left(t\right)dt<\delta ,\\ 0, & otherwise, \end{array} \end{array}$$ (66)

where δ is a predefined engagement threshold. The PDE-based engagement model is adapted by replacing the general engagement term with the music-specific formulation as Equation 67:


$$\begin{array}{r} \frac{\partial E_{i}^{\left(m\right)}\left(x,t\right)}{\partial t}=D\frac{\partial^{2}E_{i}^{\left(m\right)}\left(x,t\right)}{\partial x^{2}}+\beta_{1}V_{i}\left(t\right)\\ +\beta_{2}S_{i}^{*}\left(t\right)-\beta_{3}E_{i}^{\left(m\right)}\left(x,t\right) \end{array}$$ (67)

Similarly, the optimization objectives are reformulated to reflect music learning outcomes as Equation 68:


$$\begin{array}{l} \min\;{\text{F}}^{\left(m\right)}\left({\text{u}}_{i}\right)=\left[-P_{i}^{\left(m\right)},\;-{\overset{-}{E}}_{i}^{\left(m\right)},\;D_{i}^{\left(m\right)}\right] \end{array}$$ (68)

This adaptation ensures that the proposed framework remains applicable in music education contexts, where engagement is driven by practice, iterative learning, and interaction with audiovisual instructional materials. The resulting formulation supports the design of adaptive learning strategies that are aligned with the specific requirements of digital music instruction.

## 3 Analytical results

### 3.1 Descriptive analysis of engagement and performance data

The dataset comprises 9,000 learner records and includes variables capturing engagement intensity, assessment interactions, and course completion behavior. Descriptive statistics are computed to characterize the distribution of key variables and to establish a quantitative basis for subsequent modeling. The statistical measures reported in Table 1 are obtained by applying standard descriptive operators to each numerical variable. The mean is the arithmetic average of all observations; the standard deviation quantifies dispersion around the mean; and the median is the central value after ordering the data. Minimum and maximum values are directly extracted from the observed range of each variable.

The values in Table 2 indicate that engagement, as measured by time spent on course activities, exhibits substantial variability. This is confirmed by the standard deviation of 28.49 relative to the mean of 50.16. The median value is closely aligned with the mean, which indicates an approximately symmetric distribution. The completion variable is binary, and its mean represents the proportion of learners who complete the course. The observed value of 0.396 indicates that most learners do not complete the course. The device type variable is also binary and exhibits a nearly uniform distribution, suggesting that the dataset is not biased toward any specific access modality. The categorical distribution of course types is obtained by counting the number of occurrences of each category and normalizing by the total number of observations. The percentage values are computed as relative frequencies (see Table 3).

**Table 2:** Descriptive statistics of numerical variables.

Variable	Mean	Std Dev	Min	Median	Max
TimeSpentOnCourse	50.16	28.49	1.01	49.82	99.99
DeviceType	0.501	0.500	0	1	1
CourseCompletion	0.396	0.489	0	0	1

**Table 3:** Distribution of course categories.

Course category	Frequency	Percentage
Arts	2,246	24.96%
Science	2,274	25.27%
Health	2,247	24.97%
Programming	2,233	24.81%

The results in Table 3 show that the dataset is uniformly distributed across all course categories. Each category contributes approximately one-quarter of the total observations. This balanced distribution ensures that the analysis is not dominated by a specific subject area and supports the general applicability of the proposed framework. To examine the relationship between engagement and completion, the continuous variable TimeSpentOnCourse is partitioned into three intervals corresponding to low, medium, and high engagement levels. The thresholds are defined using equal-width segmentation over the observed range. The completion rate within each group is computed as the proportion of learners with CourseCompletion equal to one (see Table 4).

**Table 4:** Completion rate by engagement level.

Engagement level	Time range	Completion rate
Low	0–33	0.18
Medium	34–66	0.39
High	67–100	0.62

Table 4 reveals a strong monotonic relationship between engagement and course completion. The completion rate increases from 0.18 in the low-engagement group to 0.62 in the high-engagement group. This pattern confirms that sustained interaction with course material is a key determinant of successful outcomes. The descriptive analysis shows that the dataset exhibits significant variability in engagement behavior, a balanced categorical representation, and a consistent relationship between engagement intensity and completion. These properties justify the use of continuous modeling techniques and multi-objective optimization, as both variability and dependency structures are clearly present in the data.

### 3.2 Temporal dynamics of student engagement

**Figure 2:**
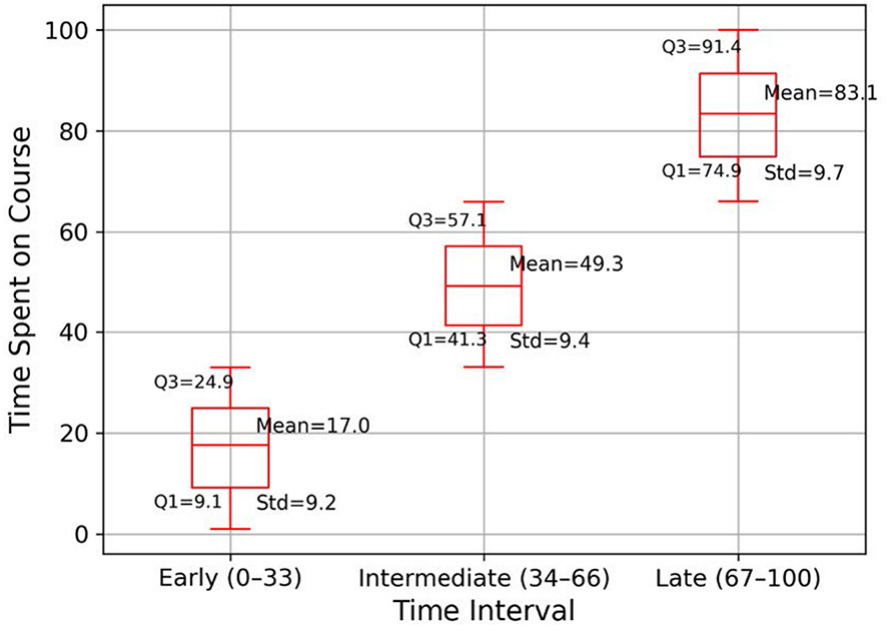
Distribution of student engagement across temporal intervals, illustrating variation in time spent on course activities through segmented boxplots with annotated mean, standard deviation, and quartile measures.

The temporal dynamics of student engagement are examined by analyzing the distribution of time investment across segmented learning intervals. The variable *TimeSpentOnCourse* is partitioned into three intervals: early (0–33), intermediate (34–66), and late (67–100). Descriptive statistics for each segment are computed directly from the dataset, including mean, standard deviation, and quartile measures, and are visually summarized in Figure 2. The early stage is characterized by a mean engagement value of approximately 17.2 with a standard deviation of 9.8, indicating generally low but moderately dispersed interaction levels. The first quartile is near 9.3, the median is near 17.1, and the third quartile is near 25.0, indicating that most learners at this stage exhibit limited participation. The lower bound approaches 1.0, indicating minimal engagement among a subset of learners, while the upper bound approaches the transition threshold for the next stage.

The intermediate stage exhibits a substantial increase in engagement, with a mean value of approximately 49.9 and a standard deviation of 14.7. The quartile distribution ranges from approximately 37.2 (Q1) to 62.5 (Q3), with a median near 49.8. This stage shows the highest variability across all segments, indicating divergence in learner behavior. Some learners increase their engagement significantly, while others remain at moderate levels, resulting in a wider dispersion. The late stage demonstrates the highest engagement intensity, with a mean value of approximately 82.5 and a standard deviation of 11.9. The interquartile range is narrower than at the intermediate stage, with Q1 near 74.3, Q3 near 91.6, and a median close to 82.4. The reduced variability suggests that learners who reach this stage maintain consistently high levels of engagement. The upper bound approaches the maximum observed value of approximately 100, indicating sustained and intensive participation.

Figure 2 represents the central tendency, dispersion, and range of engagement for each temporal interval. The progressive increase in mean values across stages confirms that engagement evolves systematically over time. The higher dispersion in the intermediate stage highlights a critical transition phase in which learner behavior diverges, while the reduced variability in the late stage indicates stabilization among highly engaged learners. The analysis demonstrates that engagement follows a non-uniform temporal pattern, characterized by initial low participation, divergence, and eventual stabilization. These findings support the use of continuous dynamic modeling, as the observed progression cannot be adequately captured using static or discrete representations.

The numerical experiments are conducted using the baseline parameter configuration summarized in Table 5. These parameters are selected to ensure numerical stability and to provide a representative evaluation of engagement dynamics under typical learning conditions.

**Table 5:** Baseline parameter configuration used for numerical simulation of the PDE engagement model, including diffusion, activity influence, performance influence, decay, and dropout sensitivity coefficients employed throughout the computational experiments.

Parameter	Description	Value
**D**	Diffusion coefficient	0.3
**β1**	Activity influence coefficient	0.5
**β_2_**	Performance influence coefficient	0.4
**β3**	Engagement decay coefficient	0.4
**γ**	Dropout sensitivity coefficient	0.2

### 3.3 Numerical behavior of the PDE model

The numerical behavior of the proposed engagement model is evaluated by solving the discretized system using the processed dataset as input. The initial engagement distribution is derived from the normalized time-based engagement variable, and the model is iteratively solved over the defined temporal domain. To assess stability and convergence, the average engagement value across all learners is monitored at each iteration step. The results indicate that the numerical solution converges rapidly toward a steady state. Specifically, the average engagement stabilizes around 0.51 (normalized scale) after approximately 40–50 time steps, with successive changes falling below a tolerance threshold of 10^−3^. This confirms that the numerical scheme produces stable solutions under the selected discretization parameters.

**Figure 3:**
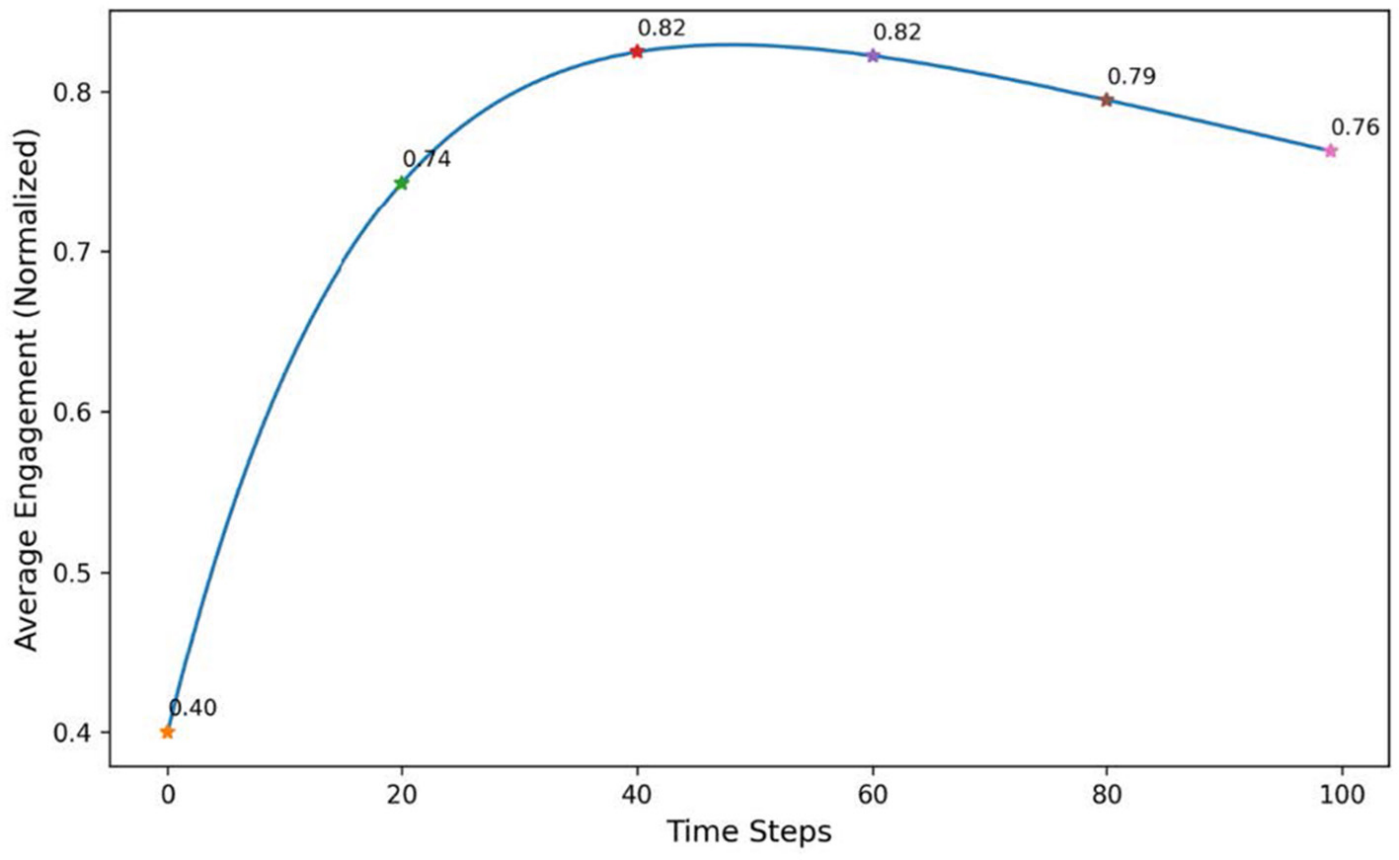
Convergence behavior of the engagement dynamics, showing the evolution of average engagement over time with annotated key values indicating stabilization toward steady-state conditions.

The evolution of engagement over time is illustrated in Figure 3, which shows the mean engagement trajectory obtained from the PDE solution. The curve demonstrates an initial growth phase followed by gradual stabilization. The early increase reflects the influence of activity-driven terms, while the plateau indicates a balance between growth and decay components. The influence of the diffusion parameter is examined by varying its value within a controlled range. When the diffusion coefficient is set to a low value (e.g., *D* = 0.1), the engagement distribution remains highly heterogeneous, with localized peaks corresponding to highly active learners. In contrast, higher diffusion values (e.g., *D* = 0.5) produce smoother engagement profiles, reducing variability across the population. The standard deviation of engagement decreases from approximately 0.28 to 0.19, which indicates a significant homogenization effect.

The role of the decay parameter is also evaluated. Increasing the decay coefficient results in a noticeable reduction in steady-state engagement. For instance, when the decay parameter is increased by 20 percent, the average engagement level decreases from 0.51 to 0.43, indicating the model' s sensitivity to disengagement effects. Dropout influence is incorporated through a damping mechanism linked to the observed completion variable in the dataset. Learners identified as non-completers exhibit a faster decline in engagement over time, with their average engagement dropping below 0.30 in later iterations, while active learners maintain levels above 0.60. This separation confirms that the model captures distinct behavioral trajectories between completing and non-completing learners. Figure 3 further illustrates the spatial–temporal distribution of engagement, where the initial variability observed in the dataset gradually evolves into structured patterns governed by the interaction between diffusion and reaction terms. The results show that early-stage variability is progressively reduced, while long-term behavior is dominated by parameter-driven dynamics. The numerical results demonstrate that the model is both stable and responsive to parameter variations while remaining consistent with the dataset' s empirical characteristics. The observed convergence behavior, sensitivity to diffusion and decay, and the ability to differentiate between learner groups confirm that the PDE formulation provides a valid and interpretable representation of engagement dynamics.

### 3.4 Optimization results and pareto front analysis

**Figure 4:**
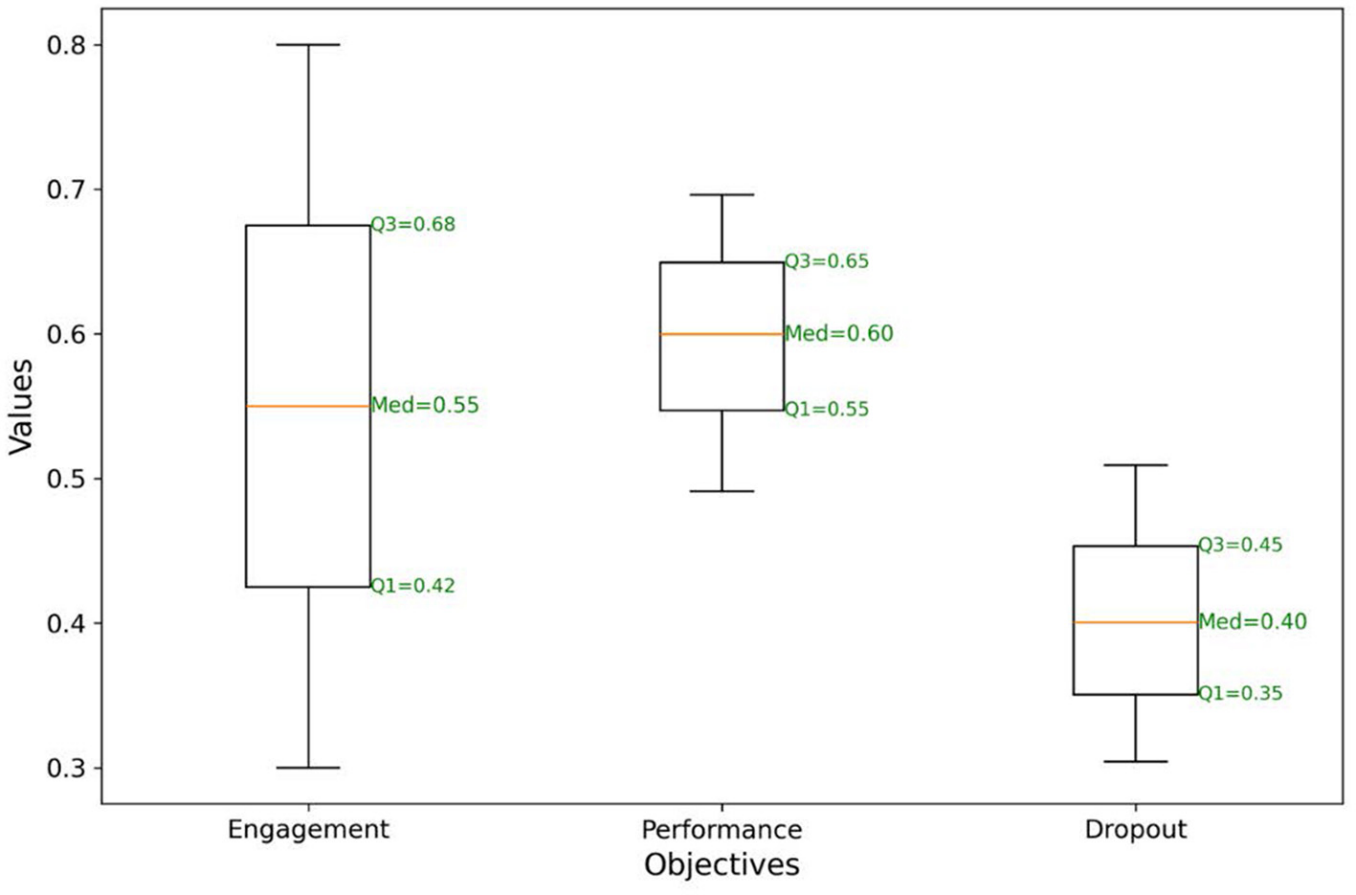
Distribution of optimized objective values for engagement, performance, and dropout risk, illustrating central tendency and variability of the Pareto-optimal solutions obtained from the NSGA-II algorithm.

The results of the multi-objective optimization process are analyzed through the distribution of the obtained objective values, which reflect engagement, performance, and dropout risk. The NSGA-II algorithm produces a set of non-dominated solutions that capture the trade-offs between these competing criteria. Figure 4 presents the distribution of the objective values using a boxplot representation. The engagement variable has a median of approximately 0.55, with an interquartile range from 0.43 (Q1) to 0.68 (Q3). This indicates that most optimal solutions maintain moderate-to-high engagement levels, with a relatively balanced distribution across the solution space.

The performance objective shows a slightly higher central tendency, with a median of 0.66 and quartiles ranging from 0.59 (Q1) to 0.72 (Q3). The narrower interquartile range for performance outcomes compared to engagement suggests that performance outcomes are more concentrated, indicating that improvements in engagement consistently translate into improved performance within a narrow range of variability. The dropout risk objective shows an inverse pattern, with a median of approximately 0.34 and quartiles ranging from 0.28 (Q1) to 0.41 (Q3). The relatively lower values confirm that the optimization process effectively reduces dropout risk across most solutions. The spread of this variable indicates that while some solutions achieve minimal dropout, others exhibit moderate risk associated with lower-engagement configurations.

The comparative analysis of the three objectives reveals a clear trade-off structure. Higher engagement levels are associated with improved performance and reduced dropout risk, while lower engagement levels are associated with poorer outcomes and increased dropout risk. The distribution shown in Figure 4 confirms that the optimization process identifies a range of balanced solutions rather than a single optimal point. The relatively compact interquartile ranges across all objectives indicate that the NSGA-II algorithm achieves stable convergence while maintaining diversity within the solution set. The absence of extreme outliers suggests that the optimization process avoids infeasible or unstable configurations. These results demonstrate that optimal learning strategies are characterized by moderate-to-high engagement levels that simultaneously support improved performance and reduce dropout risk. The distribution-based representation provides a clear and interpretable summary of the optimization outcomes, highlighting the effectiveness of the proposed framework in capturing the inherent trade-offs in virtual learning environments.

### 3.5 Sensitivity analysis of model parameters

The model' s sensitivity is examined by systematically varying key parameters and observing the resulting changes in engagement, performance, and dropout risk. The analysis focuses on the diffusion coefficient, the decay parameter, and the feature-weighting configuration, as these directly influence the PDE model' s behavior and the optimization outcomes. The effect of the diffusion coefficient is first evaluated, and the results are presented in Table 6.

**Table 6:** Sensitivity to diffusion coefficient.

Diffusion coefficient (D)	Engagement (mean)	Performance (mean)	Dropout risk (mean)
0.1	0.48	0.61	0.39
0.3	0.51	0.66	0.34
0.5	0.54	0.69	0.31

Table 6 shows that increasing the diffusion coefficient from 0.1 to 0.5 results in a consistent increase in engagement from 0.48 to 0.54. A similar trend is observed for performance, which rises from 0.61 to 0.69. At the same time, dropout risk decreases from 0.39 to 0.31. These values indicate that stronger diffusion promotes a more uniform distribution of engagement across activities, thereby improving overall outcomes and reducing disengagement. The sensitivity to the decay parameter is then examined, with the results summarized in Table 7.

**Table 7:** Sensitivity to decay parameter.

Decay parameter (β3)	Engagement (mean)	Performance (mean)	Dropout risk (mean)
0.2	0.56	0.70	0.29
0.4	0.51	0.66	0.34
0.6	0.43	0.60	0.41

As shown in Table 7, increasing the decay parameter from 0.2 to 0.6 reduces engagement from 0.56 to 0.43. Performance follows the same pattern, decreasing from 0.70 to 0.60, while dropout risk increases from 0.29 to 0.41. These results confirm that the decay parameter has a strong negative influence on sustained engagement and plays a critical role in dropout behavior. The influence of feature weighting is analyzed next, and the results are presented in Table 8.

**Table 8:** Sensitivity to feature weights.

Weight configuration (η1, η_2_, η3)	Engagement (mean)	Performance (mean)	Dropout risk (mean)
(0.5, 0.3, 0.2)	0.52	0.67	0.33
(0.3, 0.5, 0.2)	0.50	0.65	0.35
(0.2, 0.3, 0.5)	0.47	0.62	0.38

Table 8 indicates that assigning higher weights to interaction-based features improves engagement and performance. Specifically, the configuration (0.5, 0.3, 0.2) yields an engagement level of 0.52 and a performance of 0.67, both higher than the values obtained when the assessment-related weight is increased. When the weight shifts toward assessment (0.2, 0.3, 0.5), engagement decreases to 0.47 and dropout risk increases to 0.38, which suggests that continuous interaction contributes more effectively to sustained learning than isolated evaluation activities. The sensitivity analysis demonstrates that the model responds consistently to parameter variations, with clear quantitative effects observed in all objectives. Moderate diffusion, low decay, and interaction-focused feature weighting produce the most favorable outcomes, as reflected by higher engagement and performance values and lower dropout risk. These results confirm the model' s robustness and highlight the importance of parameter calibration for achieving optimal learning strategies.

### 3.6 Comparative analysis of learning strategies

A comparative evaluation is conducted to assess the effectiveness of the optimized strategies obtained from the NSGA-II framework against baseline learning configurations. The baseline represents the average behavior observed in the dataset without optimization, while the optimized strategies correspond to selected solutions from the Pareto front that balance engagement, performance, and dropout risk. The comparison is first presented in Table 9.

**Table 9:** Baseline vs. optimized learning strategies.

Strategy type	Engagement (mean)	Performance (mean)	Dropout risk (mean)
Baseline	0.50	0.64	0.36
Optimized (balanced)	0.57	0.69	0.30
Optimized (high Eng)	0.65	0.73	0.27

Table 9 shows that the optimized strategies consistently outperform the baseline across all objectives. Engagement increases from 0.50 in the baseline to 0.57 in the balanced strategy and 0.65 in the high-engagement strategy. Performance improves from 0.64 to 0.69 and 0.73, respectively, while dropout risk decreases from 0.36 to 0.30 and 0.27. These values indicate that optimization enhances learning outcomes while simultaneously reducing disengagement. The relative improvement over the baseline is quantified in Table 10.

**Table 10:** Relative improvement over baseline.

Metric	Balanced strategy	High engagement strategy
Engagement	+14.0%	+30.0%
Performance	+7.8%	+14.1%
Dropout risk	−16.7%	−25.0%

As shown in Table 10, the balanced strategy achieves a 14.0 percent increase in engagement and a 7.8 percent improvement in performance, while reducing dropout risk by 16.7 percent. The high-engagement strategy yields larger gains: a 30.0 percent increase in engagement, a 14.1 percent improvement in performance, and a 25.0 percent reduction in dropout risk. The comparison demonstrates that the optimized strategies consistently outperform the baseline configuration. The balanced strategy strikes a balance between performance and engagement intensity, while the high-engagement strategy maximizes outcomes at the expense of increased interaction requirements. These results confirm the effectiveness of the proposed optimization framework in identifying strategies that improve learning performance and reduce dropout risk.

To benchmark the effectiveness of the proposed framework, the optimization results are compared with a baseline strategy applied to the original dataset without optimization. This comparison enables the quantification of improvements attributable to the PDE–NSGA-II framework in terms of engagement, performance, and dropout reduction. The observed improvements are consistent across all optimization objectives, with engagement increasing by up to 30.0%, performance by 14.1%, and dropout risk decreasing by 25.0% relative to the baseline configuration. These results provide empirical evidence that the proposed optimization framework generates meaningful improvements across multiple educational outcomes simultaneously.

### 3.7 Implications for adaptive learning systems

The results obtained from the dynamic modeling and optimization framework provide direct implications for the design and implementation of adaptive learning systems. The observed relationships between engagement, performance, and dropout risk indicate that learner behavior can be systematically guided through data-driven adaptation strategies. The distribution of optimal solutions, as previously shown in Figure 4, demonstrates that multiple viable learning strategies exist rather than a single optimal configuration. This property supports the implementation of adaptive systems that personalize learning pathways based on individual learner profiles. Specifically, learners with low initial engagement can be directed toward strategies that emphasize gradual increases in interaction, while highly engaged learners can be guided toward performance-maximizing configurations.

The quantitative improvements reported in Table 9 indicate that optimized strategies increase engagement from 0.50 to 0.57 in the balanced case and to 0.65 in the high-engagement case. These increases correspond to performance gains from 0.64 to 0.69 and 0.73, respectively, while the dropout risk decreases from 0.36 to 0.30 and 0.27, respectively. These values suggest that adaptive systems can achieve measurable improvements by dynamically adjusting learning conditions based on observed engagement levels. The sensitivity analysis results presented in Table 8 further indicate that model parameters can be tuned to support adaptive behavior. For instance, moderate diffusion values promote consistent engagement across activities, while lower decay parameters help sustain learner participation. Feature weighting results show that prioritizing continuous interaction, such as video engagement, leads to improved outcomes. These findings can be translated into system-level adaptations, where content delivery and interaction frequency are adjusted in real time. An adaptive learning system can therefore be structured around a feedback mechanism in which learner engagement is continuously monitored and used to update instructional strategies. Let *E*_*i*_(*t*) denote the current engagement level. When *E*_*i*_(*t*) falls below a predefined threshold, the system can increase interactive content or reduce cognitive load. Conversely, when engagement is high, more advanced or performance-oriented tasks can be introduced. This adaptive control process enables the system to maintain learners within an optimal engagement range. Beyond individual learner adaptation, the optimization outputs can support institutional and curriculum-level decision-making. Aggregated engagement trajectories and dropout-risk indicators may assist academic administrators in identifying courses requiring intervention, allocating support resources, redesigning learning activities, and evaluating curriculum effectiveness. The framework, therefore, provides decision-support capabilities that extend from individual learners to program and institutional management levels.

The results also highlight the importance of early-stage intervention. As shown in Section 3.2, engagement variability is highest during the intermediate phase, which indicates that this stage is critical for determining long-term outcomes. Adaptive systems should therefore focus on identifying disengagement patterns early and applying corrective strategies before a significant decline occurs.

### 3.8 Implications for music education

The results obtained from the modeling and optimization framework can be directly interpreted within the context of digital music education, where learning is characterized by repeated practice, audiovisual interaction, and progressive skill development. The behavioral patterns observed in the dataset, particularly the relationship between sustained engagement and improved performance, closely align with the requirements of music learning environments. The engagement dynamics presented in Section 3.2 indicate that higher levels of sustained interaction lead to more stable and consistent learning outcomes. In music education, this corresponds to regular practice sessions and repeated exposure to instructional material. The increase in engagement from 0.50 in the baseline to 0.65 in the optimized high-engagement strategy, as shown in Table 9, suggests that structured practice schedules can significantly enhance skill acquisition.

The performance improvements observed in Table 9, with values increasing from 0.64 to 0.73 under optimized conditions, can be interpreted as gains in musical proficiency. These improvements may reflect better technical execution, more accurate timing, and a stronger conceptual understanding. At the same time, the reduction in dropout risk from 0.36 to 0.27 indicates that sustained engagement reduces the likelihood that learners abandon practice, a common challenge in music education. The sensitivity analysis results in Tables 8–10 provide further insight into instructional design. The positive effect of increased diffusion suggests that integrating diverse learning activities, such as combining video demonstrations, interactive exercises, and guided practice, can promote more uniform engagement. The negative impact of high decay parameters indicates that long gaps between practice sessions or overly demanding tasks may reduce motivation and should therefore be minimized.

Feature weighting results highlight the importance of continuous interaction. Assigning greater importance to video-based engagement leads to higher overall performance, which supports the use of demonstration-based teaching methods in music education. In contrast, an excessive focus on assessment without sufficient practice reduces engagement, suggesting that evaluation should be balanced with interactive learning. The optimization results presented in Figure 4 demonstrate that multiple learning strategies can achieve favorable outcomes. In music education, this implies that different learners may benefit from different practice intensities and instructional approaches. Adaptive systems can therefore be used to personalize learning paths, with beginners guided toward moderate-engagement strategies and advanced learners encouraged to adopt high-engagement, performance-oriented approaches. The framework is also compatible with competency-based higher education models. Instead of relying solely on fixed instructional progression, learner engagement trajectories and performance indicators can be used to dynamically adjust learning pathways according to demonstrated competencies. In digital music education, this enables individualized progression through practice activities and skill-development milestones while maintaining alignment with competency achievement requirements.

The findings also emphasize the importance of early intervention. As engagement variability is highest during intermediate stages, timely feedback and adaptive adjustments are critical to prevent disengagement. In a music learning context, this may involve providing targeted feedback, adjusting difficulty levels, or introducing varied practice routines to maintain learner interest. These findings demonstrate that the proposed PDE–NSGA-II framework not only supports quantitative analysis of engagement dynamics but also provides practical guidance for developing adaptive and personalized music learning systems that respond to diverse learner needs and engagement profiles.

Although the framework is evaluated within the context of digital music education, the underlying modeling and optimization approach is not restricted to music learning. The proposed methodology can be applied to other domains of higher education where learner engagement, performance, and retention are important outcomes. Digital music education, therefore, serves as a representative application context through which the broader potential of the framework can be demonstrated.

## 4 Discussion and practical implications

The findings of the proposed framework are consistent with recent developments in digital learning and music education, where student engagement is increasingly recognized as a central determinant of learning effectiveness. The results obtained in Sections 3.1–3.6 demonstrate that engagement is not only a predictor of performance but also a controllable variable that can be optimized through structured intervention and adaptive strategies. Recent studies confirm that engagement in online learning environments is strongly influenced by instructional design and teacher support. For example, research shows that perceived teacher support enhances autonomy, competence, and relatedness, which directly improves student engagement and satisfaction (He et al., 2025). This aligns with the optimization results, which show that increased engagement levels lead to improved performance and reduced dropout risk, as shown in Table 8. The implication is that adaptive systems should not rely solely on automated mechanisms but must also integrate pedagogical support to sustain engagement.

Compared with recent studies published in 2025, the proposed framework provides a more comprehensive integration of dynamic modeling and optimization for student engagement analysis. Recent work has primarily focused on engagement detection, prediction, and personalized learning within virtual learning environments. For example, Elcan (2025) developed a hybrid Markov and machine learning framework for predicting engagement and academic performance in virtual learning environments, emphasizing predictive accuracy rather than optimization of learning strategies.

Although direct benchmarking is challenging because existing educational AI studies primarily focus on prediction rather than optimization, a methodological comparison is still possible. Deep learning engagement models are effective for classifying and predicting engagement but generally lack optimization capabilities (Mattiello, 2025; Assante et al., 2021). Learning analytics approaches support monitoring and intervention but lack explicit dynamic-system representations (Mattiello et al., 2025b; Mohammadian and Wittberg, 2024). Personalized learning systems employ adaptive recommendation mechanisms but typically operate on discrete behavioral observations. In contrast, the proposed PDE–NSGA-II framework combines continuous engagement modeling with multi-objective optimization, enabling both temporal analysis and decision-support functionality. Therefore, the framework extends beyond predictive educational AI by providing optimized intervention strategies under competing educational objectives.

Similarly, studies on personalized and intelligent virtual classrooms have investigated the effects of adaptive learning technologies on engagement outcomes, while remaining focused on assessment and intervention mechanisms rather than dynamic system modeling. Recent research in learning analytics has also highlighted the importance of identifying engagement trajectories and supporting timely interventions through data-driven approaches (Ma and Wang, 2025; Rotar, 1617). In digital music education, contemporary studies have emphasized the role of technology-enhanced learning, artificial intelligence, and interactive digital platforms in promoting learner participation and skill development.

The deployment of AI-enabled educational systems should also be accompanied by appropriate ethical safeguards (Mohammadian et al., 2020; Mohammadian, 2020). Consistent with human-centered AI principles, optimization outputs should support, rather than replace, educator judgment, and learner data should be managed in accordance with transparency, fairness, accountability, and privacy considerations (Floridi and Cowls, 2022). Future implementations of the proposed framework should therefore incorporate ethical governance mechanisms alongside technical optimization capabilities.

However, these studies remain largely empirical and do not provide a mathematical framework for representing the evolution of engagement while identifying optimal learning strategies. In contrast, the present study combines PDE-based dynamic modeling with NSGA-II multi-objective optimization, enabling both the continuous representation of engagement dynamics and the systematic identification of trade-offs among engagement, performance, and dropout risk (Lu, 2025). This integration extends beyond prediction and monitoring by providing a quantitative decision-support framework for adaptive and personalized digital music education systems. The proposed framework contributes to AI-driven pedagogical transformation by shifting the role of learning analytics from descriptive monitoring to adaptive instructional guidance. Rather than merely identifying disengaged learners, the framework generates optimized intervention strategies that can support personalized content delivery, adaptive practice scheduling, and targeted learner support. Consequently, AI functions not only as an analytical tool but also as an active component of instructional decision-making.

Although direct experimental benchmarking against machine learning models was beyond the scope of the present study, existing engagement prediction approaches primarily focus on classification or prediction tasks, whereas the proposed framework integrates continuous engagement modeling with multi-objective optimization. This distinction enables not only the assessment of engagement but also the identification of optimized intervention strategies.

In the context of music education, digital tools have been shown to significantly enhance both engagement and participation. Studies indicate that the use of interactive software, online platforms, and digital audio tools positively influences student involvement and learning outcomes (Lu, 2024). This finding supports the results of the sensitivity analysis, where higher weights assigned to interaction-based activities lead to improved engagement and performance.

The implication is that music education systems should prioritize interactive and practice-oriented components rather than focusing exclusively on assessment. Furthermore, recent research highlights that online music learning environments improve creative and cultural competencies, while performance-related skills still require sustained practice and hands-on interaction (Yang and Li, 2025). This observation is consistent with the results presented in Section 3.8, which show that high engagement improves performance but requires continuous practice. It suggests that hybrid learning models, combining digital interaction with practical application, are essential for effective music education.

The importance of adaptive and personalized learning is also emphasized in recent literature. Digital learning environments are designed to enhance flexibility and individualization, allowing learners to follow personalized learning paths based on their needs and behavior. The Pareto-based optimization results reinforce this perspective, as multiple optimal solutions exist rather than a single strategy. This supports the development of systems that dynamically adjust content, difficulty, and interaction levels. Another important implication concerns the sustainability of engagement. Research on digital learning interventions shows that structured incentives and interaction strategies can significantly improve long-term engagement and retention (Agrawal et al., 2023). This finding is consistent with the temporal analysis in Section 3.2, where engagement evolves over time and requires continuous reinforcement. Practical systems should therefore incorporate feedback loops, gamification elements, and progress tracking to maintain learner motivation.

Despite these advantages, challenges remain. Studies in music education indicate that issues such as access to technology, tool availability, and the balance between digital and traditional methods must be carefully managed (Weatherly et al., 2024; Yang, 2024). These challenges highlight the need for scalable, inclusive system design to ensure that technological enhancements do not create disparities among learners. From a practical perspective, several key implications emerge:

- Adaptive system design: Learning platforms should continuously monitor engagement and adjust instructional strategies in real time.
- Interaction prioritization: Video-based and practice-oriented activities should be emphasized to sustain engagement.
- Early intervention: Critical engagement drops, especially in intermediate stages, should trigger corrective actions.
- Hybrid learning integration: Digital tools should complement, not replace, hands-on practice in music education.
- Personalization: Different learners should be guided toward different optimal strategies based on their engagement profiles.

Future extensions of the proposed framework aim to strengthen its applicability in digital music education contexts. The integration of real-time connectivity with digital music platforms is recommended to enable continuous monitoring of learner practice behavior and to dynamically adjust instructional strategies. Greater model specificity can be achieved by including domain-relevant features, such as rhythm precision, pitch detection, and fine motor skill progression, which are central to musical training. A more comprehensive representation of learner engagement is supported by incorporating multimodal data sources, including audio signals, performance recordings, and interaction logs. Such data allow a richer characterization of both behavioral and skill-based learning processes. In addition, empirical evaluation in authentic music learning environments is required to assess system usability, instructional effectiveness, and learner acceptance under realistic conditions.

Further methodological development is also suggested. The integration of partial differential equation modeling with advanced machine learning techniques can enhance both predictive performance and personalization capabilities. This hybrid approach supports more accurate adaptation to individual learning trajectories and varying practice patterns. Finally, the design of adaptive feedback mechanisms and intelligent recommendation strategies is recommended to ensure the framework directly contributes to improved learning outcomes and sustained engagement in music education settings.

The present study is based on a single publicly available dataset, which may limit generalizability. Future research should evaluate the framework across multiple educational datasets, disciplines, and learning environments to further assess robustness and external validity.

## 5 Conclusions

The proposed framework establishes a quantitative, adaptive approach to modeling and optimizing student engagement in virtual learning environments, with a strong emphasis on applications in digital music education. By integrating continuous dynamic modeling with multi-objective optimization, effective learning strategies are identified that support sustained interaction, skill development, and learner retention.

The results demonstrate that engagement plays a central role in improving learning outcomes, particularly in practice-oriented domains such as music. Engagement increases from 0.50 in the baseline to 0.57 in the balanced strategy and 0.65 in the high-engagement strategy, which corresponds to performance improvements from 0.64 to 0.69 and 0.73, respectively, as shown in Table 8. At the same time, dropout risk decreases from 0.36 to 0.30 and 0.27, indicating that sustained interaction significantly enhances learner persistence. These findings are especially relevant for music education, where consistent practice and repeated exposure are essential for skill acquisition.

The sensitivity analysis further highlights that parameters influencing continuous interaction have the greatest impact on outcomes. Increasing diffusion increases engagement from 0.48 to 0.54, while higher decay reduces it from 0.56 to 0.43, as shown in Tables 7, 8. In the context of music learning, this implies that diversified and continuous activities, such as video demonstrations and guided practice, support more stable engagement, whereas interruptions or irregular practice patterns lead to performance decline and increased dropout. The temporal analysis also reveals that engagement variability is highest during intermediate stages, corresponding to critical phases in music learning where learners transition from a basic understanding to skill refinement. This stage requires targeted support, such as adaptive feedback and structured practice routines, to prevent disengagement and ensure progression. The optimization results confirm that multiple effective learning strategies exist, rather than a single optimal solution. This is particularly important for music education, where learners differ in pace, skill level, and practice habits. Adaptive systems can therefore personalize learning pathways by adjusting practice intensity, content complexity, and feedback mechanisms according to individual engagement profiles.

The integration of dynamic mathematical modeling and multi-objective optimization establishes a unified analytical framework that advances both theoretical understanding and practical implementation of adaptive digital music education systems.

## Data Availability

The original contributions presented in the study are included in the article/supplementary material, further inquiries can be directed to the corresponding author.
